# Learning self-driven collective dynamics with graph networks

**DOI:** 10.1038/s41598-021-04456-5

**Published:** 2022-01-11

**Authors:** Rui Wang, Feiteng Fang, Jiamei Cui, Wen Zheng

**Affiliations:** 1 Institute of Public-Safety and Big Data, College of Data Science, Taiyuan University of Technology, Taiyuan, 030060 China; 2 Center for Healthy Big Data, Changzhi Medical College, Changzhi, 046000 China

**Keywords:** Phase transitions and critical phenomena, Complex networks

## Abstract

Despite decades of theoretical research, the nature of the self-driven collective motion remains indigestible and controversial, while the phase transition process of its dynamic is a major research issue. Recent methods propose to infer the phase transition process from various artificially extracted features using machine learning. In this thesis, we propose a new order parameter by using machine learning to quantify the synchronization degree of the self-driven collective system from the perspective of the number of clusters. Furthermore, we construct a powerful model based on the graph network to determine the long-term evolution of the self-driven collective system from the initial position of the particles, without any manual features. Results show that this method has strong predictive power, and is suitable for various noises. Our method can provide reference for the research of other physical systems with local interactions.

## Introduction

In the last decade, machine learning methods have excelled in the research of various scientific problems^1,2^. Therefore, various fields of natural sciences have used machine learning as a powerful tool to deal with difficult scientific problems for complex classification tasks, such as condensed matter physics^3,4^, fluid mechanics^5^, chemistry^6^ and biology^7^.

In the past few years, active matter research has also begun to successfully use machine learning methods. The progress of applying machine learning to scientific research provides new opportunities for active matter research, which enable identify optimal and alternative strategies^8^, realize the classification^9,10^ and characterization^11^ of active matter, and enable particle localization^12^ and tracking^13^. Active matter systems are not limited to thermodynamic rules such as detailed balance condition or fluctuation dissipation theorem, therefore will emerge rich and complex dynamic phenomena, such as collective motions that are widespread on the spatial scale^14–18^. Understanding the physical nature behind this phenomenon has always been an important research direction in the field of soft matter and non-equilibrium statistical physics. Reinforcement learning^19^ and deep reinforcement learning^20^ have been used to find swimming strategies that minimize energy consumption in simulated fish schools. However, traditional machine learning methods usually map graph structure data to simple representations when solving the above problems, which makes the topological information of the structure itself may be lost in the pre-processing stage and affects the final prediction results. The latest development of graph network^21^ is the promotion and extension of various previous space-based graph neural network methods. It has a strong relational inductive biases, and supports relational reasoning and combinatorial generalization. They have been used for learning and simulating complex physical systems^22–26^, computational modeling^27,28^, predicting the long-term dynamics of the glassy state^29^, and particle reconstruction^30,31^.

In this context, the study of collective dynamics deserves a systematic application of machine learning techniques in two aspects. One aspect is the description of phase transitions in non-equilibrium systems. The typical collective motion model^32^ can simulate the movement of various forms of matter, including the movement of flocks of birds and fish. Self-driven particles have a certain ability to absorb energy from the surrounding environment and somehow convert it into mechanical energy. Under the influence of the two factors of density and noise, they will transform from disordered phase to coherent phase. The phase transition process emerges spontaneous symmetry breaking process, resulting in symmetry breaking phase needs to be described by order parameter. The other is to analyze the collective phenomena and stages of physical systems with a large number of degrees of freedom. The complex collective behavior is the result of the interaction between the network topology and the laws of dynamics^33,34^. However, the nonlinear coupling relationship in the collective motion makes the complete analysis of the problem very difficult. The ability of modern machine learning technology to classify, identify, or interpret massive data sets provides new ideas for these problems: whether the basic principles of collective dynamics can be directly obtained from experimental or simulated data.

In this work, we simulated the self-driven collective motion under different noise and density according to the principle of Vicsek^32^. Through simulation we find that the motion state of the collective is accompanied by the generation and change of clusters, so we propose a cluster order parameter *k*, that is, the number of clusters divided in the collective, to quantify the degree of synchronization of the system. Obviously, the self-driven multi-individual system forms a dynamic network, and some basic concepts in graph theory are helpful for problem analysis. We processed simulated data into a graph structure and constructed the Collective Dynamics Graph network (CDGNet) model, which uses graph networks^21^ to reason about objects and relationships in self-driven collective systems, so as to accomplish long-term prediction of system order parameters. The advantage is that it fully considers the relationship between individuals and neighbors from the perspective of network topology.

## Results

### Data generation

The Vicsek model^32^ is a mathematical model used to describe active matter, which exhibits collective motion under higher particle density or lower noise. According to the rules of the Vicsek model, we use MATLAB to design iterative procedures to simulate the self-driven collective dynamics of $N = 4000$ particles in a box with two-dimensional periodic boundary conditions. We set its initial speed to 0.03, $\rho$=4, and the noise $\eta$ changes from 0 to 5. $\eta$ represents the disturbance in the environment, which will affect the direction update of particles, see “Methods” section for details. At the same time, we simulated the initial speed of 0.03, $\eta$=2, and the change of density from 0.1 to 10 to analyze the influence of noise and density on order parameters respectively.

Further, in order to train the CDGnet model, we choose the case where $N=4000$, $\rho$=4, and $\eta$=0.1, 1.5, 2.5, 3.5, 5. Our goal is to train the model under five noise points, covering different noise and time steps. For each point, we generate 100 independent configurations as training examples of the network and augmented the data, followed by generating a test set of 100 independent configurations to evaluate the model. In each independent data set, we use different initial particle positions and initial motion angles to simulate the motion of the self-driven collective, and obtain the particle position and motion angle after each iteration. The total number of iterations under different noise points is determined by the time it takes the system to reach a steady state.

### A new cluster order parameter

It can be seen from the update formula of the Vicsek model that noise and density are two important factors that affect the evolution of the collective, whose definition is detailed in “Methods” section. Therefore, in order to study the evolutionary behavior of the self-driven collective system, we simulated the conditions of different densities and noises, and got some meaningful results.

**Figure 1:**
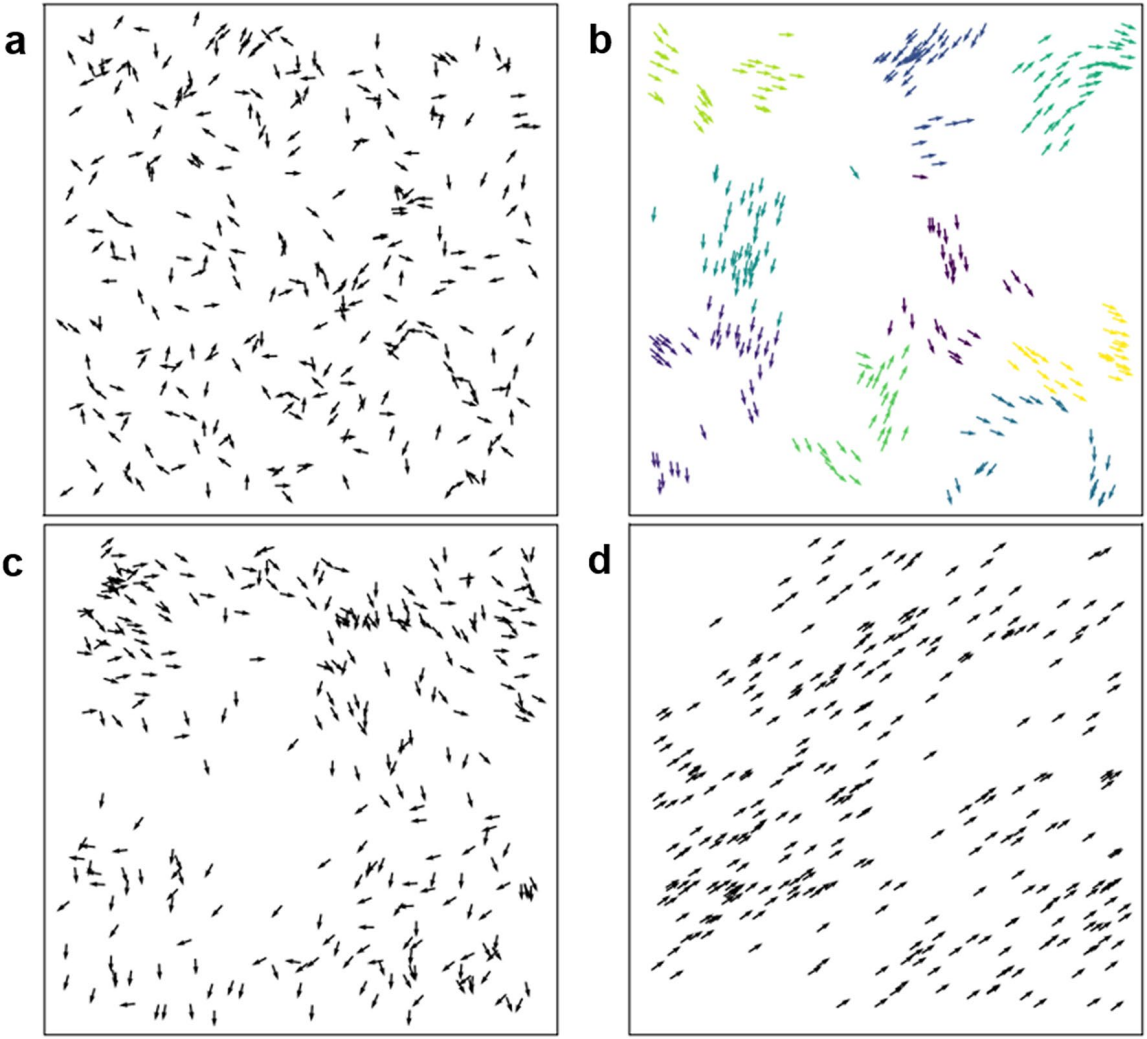
Simulation of collective motion at different densities and noises. The actual speed of the individual is indicated by a small arrow, and the tail of the arrow is the actual position of the particle. In each case, the number of particles is N = 300. (**a**) In the initial state of *L* = 7 and $\eta$ = 2.0, the position and direction of the particles are random. (**b**) In the case of low density and low noise (*L* = 25, $\eta$ = 0.1), the particles in the collective will aggregate after a period of time to form small clusters, where different colors indicate that the particles belong to different clusters. (**c**) For high-density and high-noise (*L* = 7, $\eta$ = 2.0), the movement direction of the particles in the collective is random in a small range, but there is a certain correlation overall. (**d**) In the case of high density and low noise (*L* = 5, $\eta$ = 0.1), the individuals in the collective move in the same direction. All results are obtained from simulations with a rate set to 0.03.

In Fig. 1a–d, the location and speed information during collective evolution is presented by setting different densities and noises. (a) At the initial moment, individuals are randomly distributed on a two-dimensional plane and move in random directions, and the entire collective exhibits a disordered state. (b) For low density and low noise, small clusters moving in random directions are formed in the collective. (c) In the case of high density and high noise, random motions with a certain correlation appear among the particles. (d) At low noise and high density, the collective has obtained meaningful results after evolution which is that all individuals move in the same direction. That is to say, the individuals in the collective will eventually reach synchronization after a finite movement time (convergence time).

**Figure 2:**
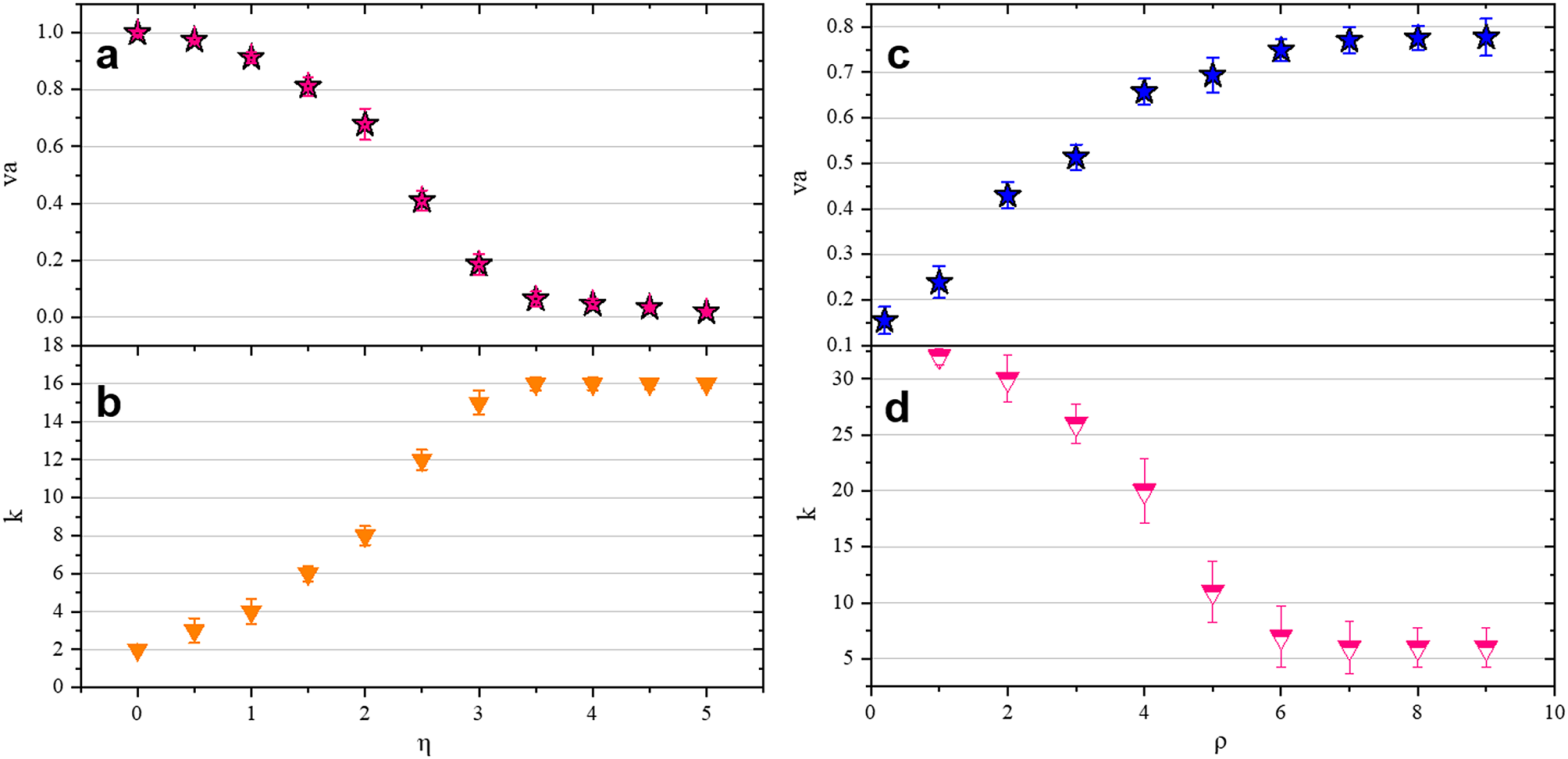
The effect of noise and density on order parameters. (**a**) The density is the same, (N = 4000, L = 31.6, that is, $\rho$ = 4) the relationship between $v_a$ and noise, $v_a$ decreases with the increase of noise. (**b**) When the density is the same (*N* = 4000, *L* = 31.6, $\rho$ = 4 can be obtained), the relationship between *k* and noise in the collective. When the density is constant, as the noise increases, the value of *k* increases accordingly. (**c**) In the case of constant noise (here, $\eta$ = 2.0, L = 20), $v_a$ increases with the increase of density. (**d**) When the noise is constant ($\eta$ = 2.0, L = 20), the relationship between *k* and the density in the collective. As the density increases, the *k* value decreases.

In the simulation, we found that some clusters will be formed during the evolution of the collective. How to divide clusters is an important research question. How to divide clusters is an important research question. Peruani et al.^35^ define the two neighboring individuals as members of the same cluster if their centers of mass is less than *R*, and their motion directions differ by less than the angle $\alpha$. This method relies on the choice of parameters *R* and $\alpha$ for the definition of clusters. Different parameter combinations will lead to different results, and the process of parameter selection is more complicated. Chen et al.^36^ used the Canny-Deriche algorithm detect the boundaries of cell clusters in clustering images, and divided the areas of the clusters in pixels. The number of individuals in a cluster is the area of a cluster divided by the average area covered by a single cell. The method does not consider the motion direction of individual when dividing clusters. Therefore, we designs an algorithm based on the k-means algorithm to calculate the optimal number of clusters. It only needs to input the position and motion direction information of the individuals in the system to automatically calculate the number of clusters *k*. k-means is a basic algorithm for partitioning the number of known clustering categories. It divides the spatial distance index of the research object into several subsets according to the similarity criterion, so that the difference between the elements in the same subset is the smallest, and the difference between the elements in the different subsets is the largest. Obviously, if the position and direction of particles in the self-driven collective dynamics system are used for clustering, the higher the consistency of collective movement direction and the closer the distance between particles, the smaller the difference between individuals, so the *k* value should be small.

The cluster validity index is usually used to evaluate the clustering results, and the number of clusters corresponding to the optimal clustering results is regarded as the optimal number of clustering categories. Some indicators have been proposed to test the effectiveness of clustering^37–39^. In this paper, the average contour coefficient is used to evaluate the clustering results, whose definition is detailed in “Methods” section. The value range of the average contour coefficient *S* is [− 1,1], and the closer the distance between the samples in the cluster, the farther the sample distance between the clusters, the larger the average contour coefficient, the better the clustering effect. Then, the *k* with the largest average contour coefficient is the optimal number of clusters.

Based on the validity index and clustering algorithm, this paper designs an algorithm to calculate the optimal number of clusters *k*, see “Methods” section for details. We calculated the changes of *k* with noise and density respectively, and compared and analyzed the results with traditional order parameter $v_a = \dfrac{1}{Nv}\mid \sum _{i=1}^{N}{\mathbf{v}}_i \mid$ (Fig. 2a–d). Changing the noise at a fixed density, we can observe the transition of particles from the disordered motion phase to the coherent motion phase. It can be seen from results in Fig. 2a that when the noise $\eta$ in the environment gets smaller, the value of $v_a$ will be closer to 1, that is, the degree of synchronization of the collective is higher. This conclusion is consistent with the assumption and analysis in Methods. As the noise increases, the degree of order decreases, that is, the system can finally reach the synchronization state only under low noise conditions. At the same time, the smaller the noise in the environment, the smaller the value of *k* (Fig. 2b). This is because the movement directions between particles are similar, so when the method described above is used to calculate the cluster order parameter *k* based on similarity criterion, the obtained value is smaller.

Density is another important factor affecting the evolution of the collective. In order to find out the influence of density, we gradually increase the system density under the premise of fixed system noise and simulated box side length. In Fig. 2c,d, we show how $v_a$ and *k* will change when the noise remains constant and the density changes. When the density of the entire system is low, $v_a$ is zero, and the system is a disordered phase; when the particle density is high, $v_a$ is non-zero, and the system is an ordered phase. At this time, the rotational symmetry of the system is broken and there is an overall non-zero mean pointing. It is worth noting that when the particle density is high, the similarity of the position and movement direction between particles also increases. Therefore, when the method described above is used to calculate the optimal number of clusters *k* based on distance clustering, the obtained value is small, which is consistent with the original idea. The quantitative analysis of the noise and density in the system proves the rationality of the order parameters proposed in this paper.

**Figure 3:**
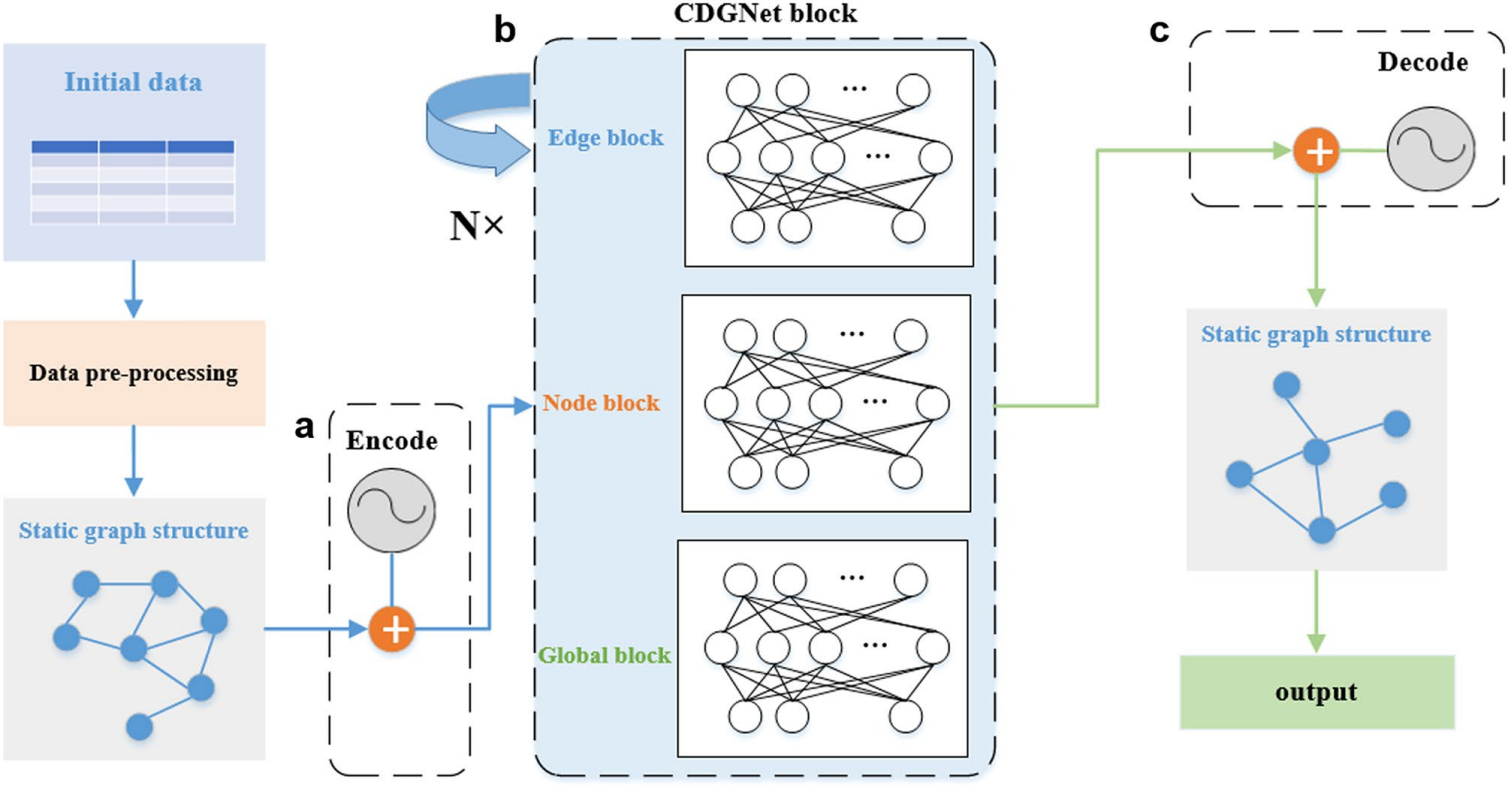
The effect of noise and density on order parameters. (**a**) The density is the same, (N = 4000, L = 31.6, that is, $\rho$ = 4) the relationship between $v_a$ and noise, $v_a$ decreases with the increase of noise. (**b**) When the density is the same (*N* = 4000, *L* = 31.6, $\rho$ = 4 can be obtained), the relationship between *k* and noise in the collective. When the density is constant, as the noise increases, the value of *k* increases accordingly. (**c**) In the case of constant noise (here, $\eta$ = 2.0, L = 20), $v_a$ increases with the increase of density. (**d**) When the noise is constant ($\eta$ = 2.0, L = 20), the relationship between *k* and the density in the collective. As the density increases, the *k* value decreases.

**Figure 4:**
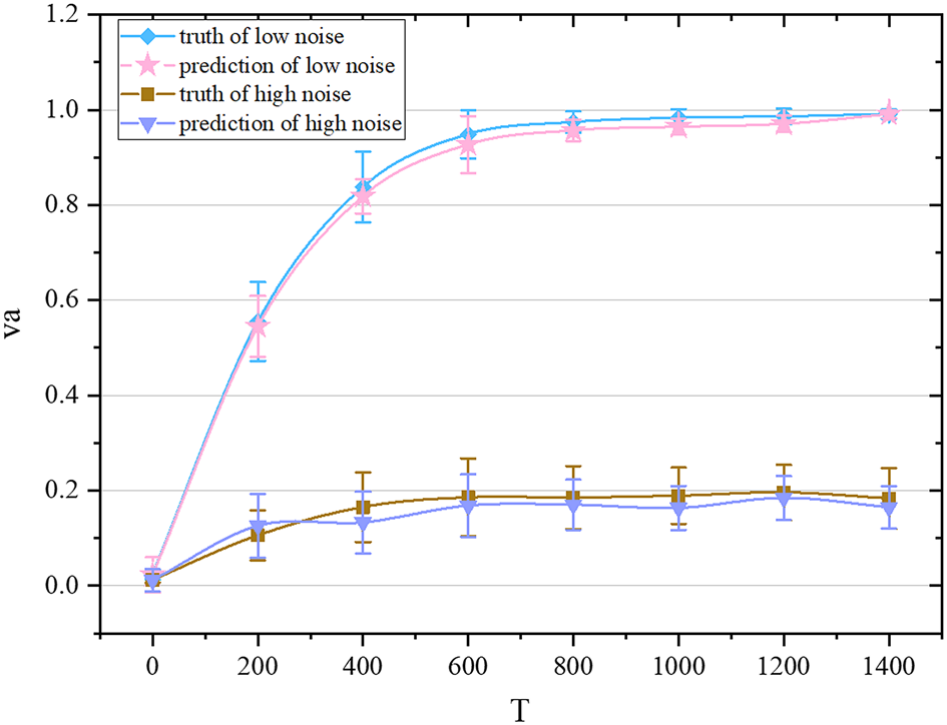
The truth and prediction of $v_a$ over time under different noises. The diamond represents the actual value of $v_a$ for N = 4000, L = 31.6, $\eta$ = 0.1, the pentagram represents the predicted value of $v_a$ for N = 4000, L = 31.6, and $\eta$ = 0.1; the square represents the actual value of $v_a$ for N = 4000, L = 31.6, $\eta$ = 3.5, and the inverted triangle represents the prediction value of $v_a$ for N = 4000, L = 31.6, and $\eta$ = 3.5.

### Prediction of order parameters under various noises

While constructing the data set, we calculated the $v_a$(*k*) of the system after each iteration, and trained the network of this paper to predict the change of $v_a$(*k*). For each training example, the positions and motion angles of N particles are put into the CDGNet model, whose definition is detailed in “Methods” section. The graph is constructed based on the distance between particles: since the field of view radius of the simulated data is 1, two particles whose distance is less than this threshold are connected by the edge. The motion angle of the particles is the attribute of the node, the relative distance between the particles is the attribute of the edge, while $v_a$(*k*) after each iteration is the global attribute. We apply a CDGNet block to this input graph to predict $v_a$(*k*). The CDGNet block uses three Multi-Layer Perceptrons(MLP) to independently input the nodes, edges, and global attributes of the graph, as shown in Fig. 3b. Then, message passing on this graph, recursively update the edges, nodes, and global of the graph. The update of each edge according to the given edge, node, and global attributes, which is connected and realized through the edge MLP. Node is updated based on the given node attributes and the aggregation of its associated edge attributes, as well as the influence of global attributes. Similarly, all nodes are updated in parallel using the node MLP. Finally, global is updated through the global MLP based on the attributes of the given global and the aggregation of all nodes and edges.

In the whole process of model construction, the input data is preprocessed, and the particle information in self-driven collective dynamics is transformed into a graph structure. The node, edge, and global MLPs receive attributes from the encoded graph (Fig. 3a) as input. In the CDGNet block, it is updated n times in a loop (we set n = 7) to ensure that the information of each particle is transmitted to the edge of the periodic box. The network decodes(Fig. 3c) the generated embedding into a predicted $v_a$(*k*), which is returned to the target $v_a$(*k*) via random gradient descent.

With a fixed density, we predict the change of $v_a$ with time in the low and high noise cases respectively. As can be seen in Fig. 4, our network achieves very good results. At a low noise level ($\eta = 0.1$), the statistical dispersion of $v_a$ is greater in the early stage of collective evolution. As the entire collective becomes stable, $v_a$ fluctuates smaller and smaller, as the different simulation situations have the same law in the stable phase: $v_a$ approaches 1. $v_a$ changes from 0 to 1, which means that the collective has completed the transition from the disordered phase to the ordered phase. When the noise is large ($\eta = 3.5$), the statistical dispersion of the value of $v_a$ in the stable phase is reduced, but it still fluctuates within a certain range. $v_a$ finally stabilizes to about 0.2. It shows that in the case of high noise, the individuals in the self-driven collective will not eventually move in the same direction. After a period of evolution, the entire system can reach a relatively stable state, but the movement directions of the particles are different.

**Figure 5:**
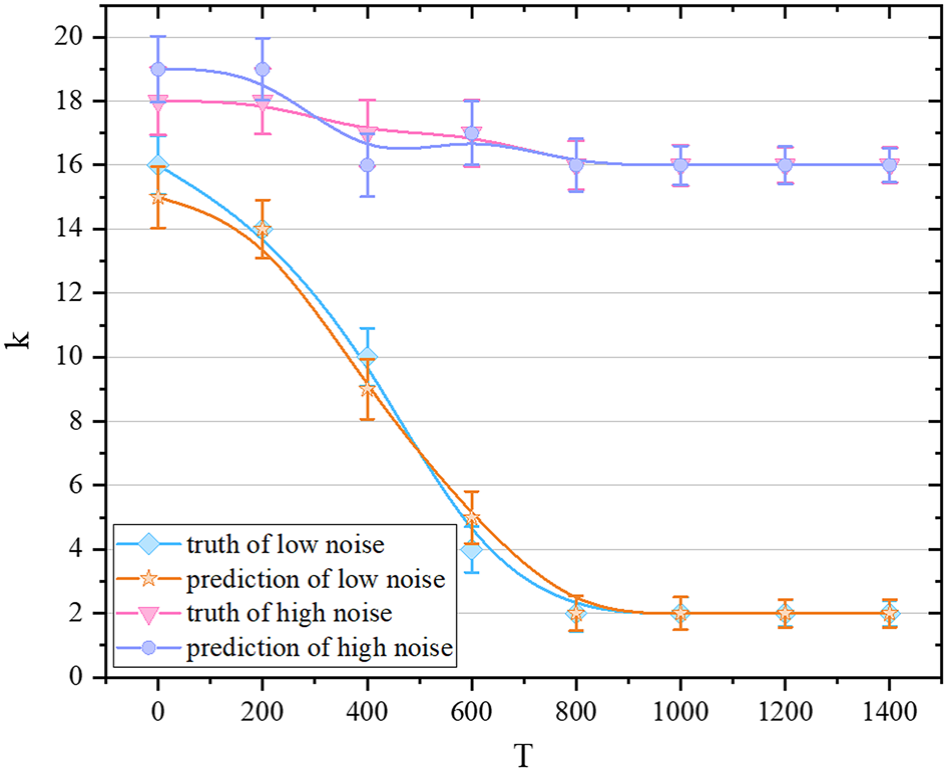
The truth and prediction of the cluster order parameter over time under different noises. The diamond represents the actual value of the cluster order parameter *k* when N = 4000, L = 31.6, and $\eta$ = 0.1, the pentagram represents the predicted value of the cluster order parameter *k* when N = 4000, L = 31.6, and $\eta$ = 0.1; the inverted triangle represents the actual value of the cluster order parameter *k* when N = 4000, L = 31.6, and $\eta$ = 3.5, the circle represents the predicted value of the cluster order parameter *k* when N = 4000, L = 31.6, and $\eta$ = 3.5.

**Figure 6:**
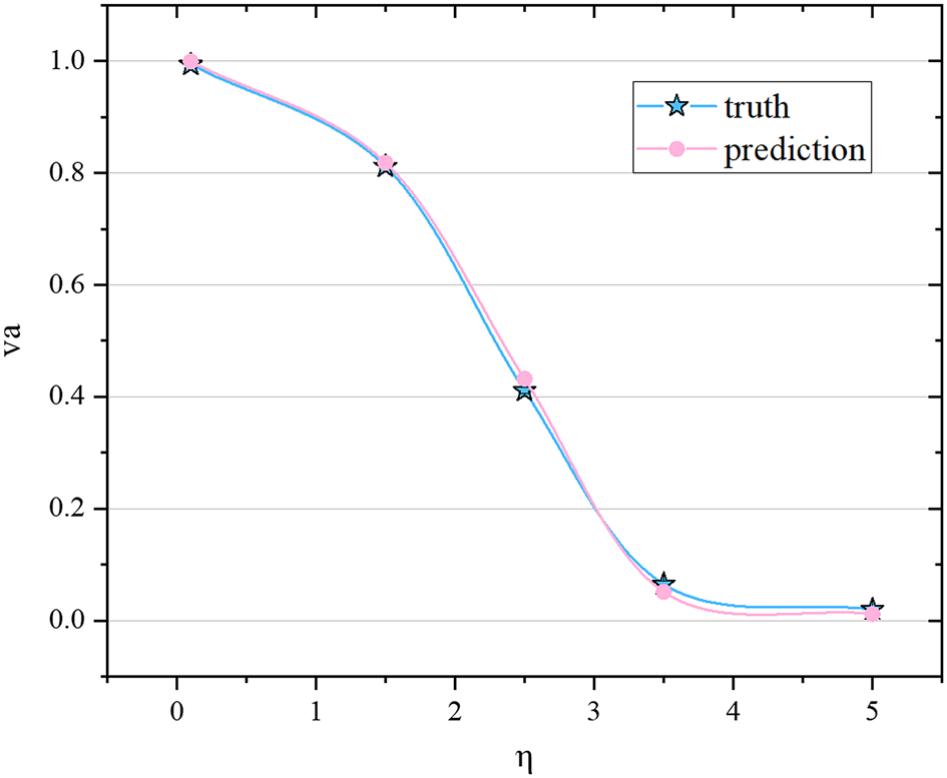
The truth and prediction of $v_a$ varying with noise. The pentagram represents the actual value of $v_a$ for N = 4000 and L = 31.6, the circle represents the predicted value of $v_a$ when N = 4000 and L = 31.6.

We respectively predict the cluster order parameter *k* at different time scales in high and low noise, as shown in Fig. 5. When the noise is small ($\eta$ = 0.1), in the early stage of collective evolution, the position and movement trend of particles differ greatly between different simulation situations, which makes the standard deviation of *k* value larger, and the whole collective is more chaotic at this time, so the prediction accuracy of the *k* value will be lower than that of the stable phase. When the noise is large ($\eta$ = 3.5), the prediction and truth of *k* will fluctuate throughout the evolution process, and the amplitude is greater than that in the case of low noise. This is because increasing the disturbance will affect the information that tends to be synchronized between the particles. However, from an overall point of view, our model can make more accurate predictions for the cluster order parameter of different time scales under different noise levels, so as to assess the degree of collective consistency in the evolution process.

To further investigate the influence of noise on the evolution of self-driven collective, we train the model at different noise points to predict $v_a$. It can be seen that our network has a strong predictive ability, with high accuracy from low to high noise predictions, as shown in Fig. 6. Results show that the noise in the environment has a significant effect on the evolution of the self-driven collective. If there is no disturbance, the particles in the collective will move in the same direction, and the entire collective will be in a synchronized state. As the disturbance in the environment increases, the collective movement will change to a disordered phase.

**Figure 7:**
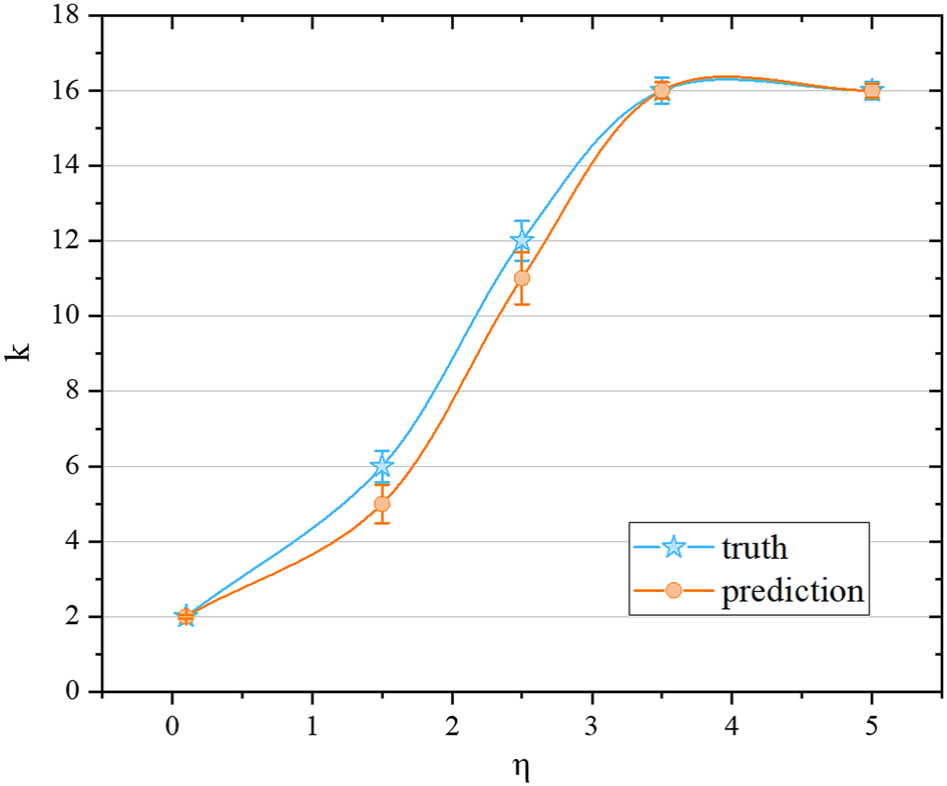
The truth and prediction of *k* varying with noise. The pentagram represents the actual value of *k* when N = 4000 and L = 31.6, the circle represents the predicted value of *k* when N = 4000 and L = 31.6.

At the same time, we train the model under different noise conditions to predict the cluster order parameter *k* at steady state. Figure 7 shows that under certain noise levels, the standard deviation of the *k* values at stable stage is large, which reduces the prediction accuracy of the model. However, as a whole, our model can make accurate predictions of the cluster order parameter under different noise to evaluate the degree of during synchronization during the phase transition.

**Figure 8:**
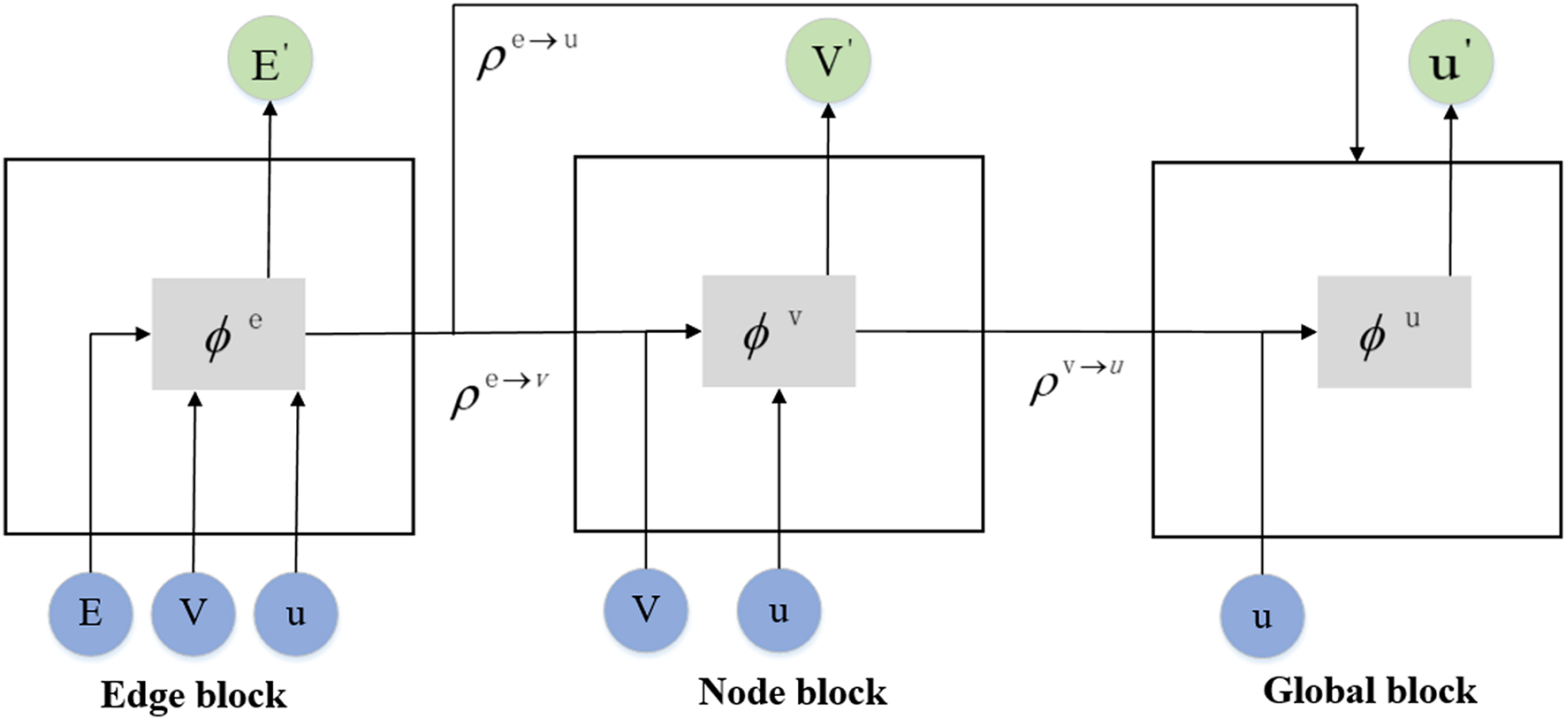
The basic building blocks of a graph network. It is divided into three main blocks: edge, node, and global. Information is disseminated and updated through certain rules.

## Discussion

The formation of clusters is a very meaningful phenomenon in the process of collective evolution, in recent years, the research of aggregation phenomenon in collective dynamics has attracted many attention^40,41^. In the process of simulating the Vicsek model, we found that the change of collective state will be accompanied by the generation and change of clusters. Therefore, it is expected that the number of clusters can be used to describe the phase transition of the collective. This paper proposes cluster order parameter *k*, and have proved the rationality of *k* through quantitative analysis.

In some cases, compared with the traditional $v_a$, the cluster order parameter *k* has more advantages. One is that the cluster order parameter *k* is calculated based on the k-means method in machine learning. The algorithm uses the information of particle position and movement direction, and divides the particles in the system into k sets according to the similarity criterion. This way of describing the collective phase transition is more intuitive, as shown in Fig. 1b. The second is that the cluster order parameter *k* can distinguish the collective state in some special cases. such as Fig. 1b,c in the paper. These two figures are the movement conditions of the collective in different environment. Obviously, the aggregation of them shows different characteristics. The system in Fig. 1b appears as small clusters, while the particles in Fig. 1c move randomly over a small area, but with some overall correlation, forming a large cluster. These differences cannot be shown through $v_a$, which focuses more on quantifying the uniformity of the motion direction of the system. The closer to 1 the higher the degree of synchronization of all particles. However, by calculating *k*, the number of clusters in the system can be obtained, so as to distinguish collective states with different characteristics.

At the same time, we built a graph network model based on deep learning to achieve long-term prediction of self-driven collective dynamics, taking advantage of the structure hidden in the local neighborhoods of the particles, and training the model by learning the interactions between the particles to predict the values of the order parameter. In this way, it is possible to quickly predict the order parameters of the collective without simulating the dynamics of the self-driven collective, thereby quantifying the degree of synchronization of the entire collective.

In conclusion, we believe that this work shows the potential of using graph network methods for exploration in the field of soft matter. In addition to the formation of dynamic clusters, many other dynamic patterns have been formed in the active matter system, such as dynamic hyperuniform^42–44^ and motility-induced phase separation^45,46^, which is the direction we will explore in future work.

## Methods

### Vicsek model

In the Vicsek model^32^,*N* individuals that can be regarded as mass points move at the same rate on the plane of the $L*L$ two-dimensional periodic boundary condition. At the initial moment, the position of each individual is randomly distributed in the plane area, and the direction of the movement of each individual is randomly distributed between $[-\pi ,\pi )$.

At each time $t+1$, the angle of each individual is updated according to the vector average of the neighbor angles, and some random disturbances ($\eta$) are added. The neighbors of an individual i consist of individuals centered at the current position of that individual and whose distance from that individual is less than the radius r of the field of view.

Each individual always moves at *a* constant rate *v* in the plane, so the equation for position change is:

1 $$\begin{aligned} {\left\{ \begin{array}{ll} x_i (t+1)=x_i (t)+vcos\theta _i (t)\Delta t\\ y_i (t+1)=y_i (t)+vsin\theta _i (t)\Delta t \end{array}\right. } i=1,2,\ldots ,N \end{aligned}$$

where $\theta _i (t)$ is the angle at which individual *i* moves at moment *t*, and its update rule is:

2 $$\begin{aligned} \theta _i (t+1)=<\theta _i(t)>_r+\Delta \theta \end{aligned}$$

where $\Delta \theta$ represents the random number chosen with uniform probability from the interval $[-\eta /2,\eta /2]$, and $\eta$ represents the noise in the environment. $<\theta _i (t)>_r$ is the average movement direction of all individuals (including individual *i* itself) within the field of view radius *r* with individual *i* as the center. It is calculated by the following formula:

3 $$\begin{aligned} <\theta _i(t)>_r=tan^{-1}\left[ \dfrac{\sum _{j\in N_i(t)}sin\theta _j(t)}{\sum _{j\in N_i(t)}cos\theta _j(t)}\right] \end{aligned}$$

Where $N_i (t)$ represent the neighbor of individual *i* at time *t*.

To analyze the synchronization of the model, Vicsek et al. define an order parameter called the absolute value of the average normalized velocity, denoted by $v_a$, which is defined as:

4 $$\begin{aligned} v_a=\dfrac{1}{Nv}\mid \sum _{i=1}^{N}{\mathbf{v}}_i \mid \end{aligned}$$

In this way, $v_a$ can be used to characterize the degree of synchronization of all individuals in the collective. If after several time steps, all individuals in the collective reach a synchronized state, the value of $v_a$ approaches 1 at this time; if the movement direction of all individuals in the collective is random, then the value of $v_a$ is approximately 0. Obviously, 0$\le v_a \le$1, larger values indicate greater consistency in the direction of individual movement. When $v_a$ = 1, all individuals move in the same direction.

### Definition of the average contour coefficient

The contour coefficient of a sample point $X_i$ is defined as follows:

5 $$\begin{aligned} S_i=\frac{b-a}{max(a,b)} \end{aligned}$$

where *a* is the average distance between $X_i$ and other samples in the same cluster, called the cohesion degree, and *b* is the average distance between $X_i$ and all samples in the nearest cluster, called the separation degree. The definition of ${X_i}$’s nearest cluster is as follows:

6 $$\begin{aligned} C_j=\mathop {\arg \min }_{C_k}\frac{1}{n}\sum _{p\epsilon C_k}{\mid p-X_i \mid }^2 \end{aligned}$$

where *p* is a sample in a certain cluster $C_k$, and *n* is the number of samples in $C_k$. In fact, after using the average distance of $X_i$ to all samples of a certain cluster as a measure of the distance from the point to the cluster, the cluster with the smallest distance from $X_i$ is selected as the closest cluster.

After calculating the contour coefficients of all samples, average the contour coefficients to obtain the average contour coefficient. Calculated as follows:

7 $$\begin{aligned} S=\frac{1}{n}\sum _{i=1}^nS_i . \end{aligned}$$

### Algorithm for finding the optimal number of clusters

The search range of the number of clusters is $[k_{min},k_{max}]$, $k_{min}$=1 means that the sample is evenly distributed without obvious feature differences. Usually, the minimum number of clusters is 2. For how to determine $k_{max}$, most scholars use empirical rules^38^: $k\le \sqrt{n}$.

The average contour coefficient index described above has a good test performance due to its simple structure and low computational complexity. Based on this, this paper designs a spatial clustering *k*-value optimization algorithm. The algorithm process is described as follows:

Algorithm: Based on the *k*-means algorithm, the optimal *k* value is calculated by the average contour coefficient.

Input: Data containing n objects.

Output: *k* value under the maximum condition of the average contour coefficient.

Algorithm steps:

1. Calculate the upper bound of the optimal solution $k\le \sqrt{n}$;
2. Use the k-means algorithm to achieve spatial clustering under all numbers when $k\le \sqrt{n}$, where the position and direction of the particles are used as the feature vector of k-means clustering, feature list = [$x,y,v_x,v_y$].;
3. Calculate the *S* value under different cluster numbers *k* according to the average contour coefficient method;
4. Search for the largest average contour coefficient *S*, and write down the corresponding *k*;
5. End.

### The CDGNet model

Graph networks^21^, a generic modular framework for deep learning, can be viewed as a superset of previous graph-based neural networks. The advantage of GN is its versatility, allowing it to be used for analysis if the structure of the target problem can be encoded in the form of a graph, or if a priori knowledge of the relationships between input entities can itself be described as a graph. GN also has strong combination and generalization capabilities. The GN block supports depth or recursive arrangement. Information can be propagated across the graph network to allow more information to perform computations and complex functions. At the same time, the calculation is not performed at the macro level of the entire system but is multiplexed across entities and relationships, which enables the network to have a good fitting result for unknown data sets. Here, we will outline the implementation of the CDGNet model for a self-driven collective.

GN is a graph-to-graph function which can be represented by $G=(u,V,E)$, with *V*, *E*, and *u* for the node (individuals), edge (effects between individuals), and global attributes respectively. For a self-driven collective system, this graph is constructed based on the distance between individuals, and edges are defined by the relative distance between particles. The set of nodes is $V=\{v_i\}_{i=1:N^v}$, where $v_i$ denotes the attribute of the ith node, $N^v$ is the number of nodes. The set of edges is $E=\{(e_k,r_k,s_k)\}_{k=1:N^e}$, where $e_k$ represents the attribute of the $k_{th}$ edge, $r_k$ and $s_k$ are the indexes of the nodes connected by the kth edge, which are the receiving node and the sending node respectively, and $N^e$ is the number of edges.

A GN block contains three blocks: edge, node, and global. These blocks can be composed of deep or recurrent neural network configurations. The blocks are related through certain rules to update the graph, as shown in Fig. 8. It includes six internal functions, three update functions, and three aggregate functions.

The update rules for the GN block are as follows:

First, the edge block calculates an output $e_k^\prime$ for each edge, and updates it with attributes from itself, its connected individuals (with indexes $v_{r_k}$ and $v_{s_k}$), and the global attribute *u*, as follows:

8 $$\begin{aligned} e_k^\prime =\phi ^e (e_k,v_{r_k} ,v_{s_k},u) \end{aligned}$$

Among them, $\phi ^e$ is the update function of the edges. Next, for each node, the node block aggregates all edges pointing to it into $\bar{e}_i^\prime$, as follows:

9 $$\begin{aligned} \bar{e}_i^\prime =\rho ^{e\rightarrow v} (E_i^\prime ) \end{aligned}$$

where $\rho ^{e\rightarrow v}$ is the aggregate function of directed edges pointing to each receiving node, $E_i^\prime =\{(e_k^\prime ,r_k,s_k)\}_{r_k=i,k=1:N^e}$ is the set of all directed edges pointing to the node with index i.

Then the output $v_i^\prime$ of each node is calculated. The attributes of each node use its own attributes, the edges connected to it, and the global attribute *u* to update:

10 $$\begin{aligned} v_i^\prime =\phi ^v (\bar{e}_i^\prime ,v_i,u) \end{aligned}$$

Among them, $\phi ^v$ is the update function of the node.

Finally, in the global block, the output at the edge and node levels are aggregated to calculate global attributes:

11 $$\begin{aligned} \bar{e}^\prime= & {} \rho ^{e\rightarrow u}(E^\prime ) \end{aligned}$$

12 $$\begin{aligned} \bar{v}^\prime= & {} \rho ^{v\rightarrow u}(V^\prime ) \end{aligned}$$

13 $$\begin{aligned} u^\prime= & {} \phi ^u(\bar{e}^\prime ,\bar{v}^\prime ,u) \end{aligned}$$

Among them, $\rho ^{e\rightarrow u}$ is the aggregate function of all edges on the graph, all the updated edges are aggregated into $\bar{e}^\prime$, where $E^\prime =\{(e_k^\prime ,r_k,s_k)\}_{k=1:N^e}$. $\rho ^{v\rightarrow u}$ is the aggregate function of all nodes on the graph, which aggregates all updated nodes as $\bar{v}^\prime$, where ${V}^\prime =\{{v_i}^\prime \}_{i=1:N^v}$. $\phi ^u$ is the update function of the global attributes. Therefore, the output of GN is the collection of all edge, node and graph-level attributes, $G^\prime =(u^\prime ,V^\prime ,E^\prime )$.

The choice of update function $\phi ^e$, $\phi ^v$ and $\phi ^u$ directly determines the performance of the model in actual tasks. In the CDGNet model, we chose Multi-Layer Perceptron (MLP) as the update function as shown in Fig. 3b. For feature extraction and data downscaling, an encoder was added to preprocess the inputs before the CDGNet block. It is found that this method improves the training speed and accuracy of the model. In the CDGNet block, three multilayer perceptrons are used to update node, edge and global attributes, respectively. The CDGNet block loops n times to implement complex calculations, and the processed information is decoded with a decoder into the desired form. The encoder compresses the input into the latent space representation, which can be represented by the function *f*(*x*), and the decoder reconstructs the latent space representation into the output, which can be represented by the function *g*(*x*), the encoding function *f*(*x*) and the decoding function *g*(*x*) are all neural network models.

## Supplementary Information

Supplementary Information.
