# Supplementary material for: Learning self-driven collective dynamics with graph networks
Source: Sci Rep. 2022 Jan 11;12:500. doi: 10.1038/s41598-021-04456-5 (PMC8752591; doi:10.1038/s41598-021-04456-5)
Supplement: Supplementary file 1 — Supplementary Information. [file 41598_2021_4456_MOESM1_ESM.pptx]

## Slide 1
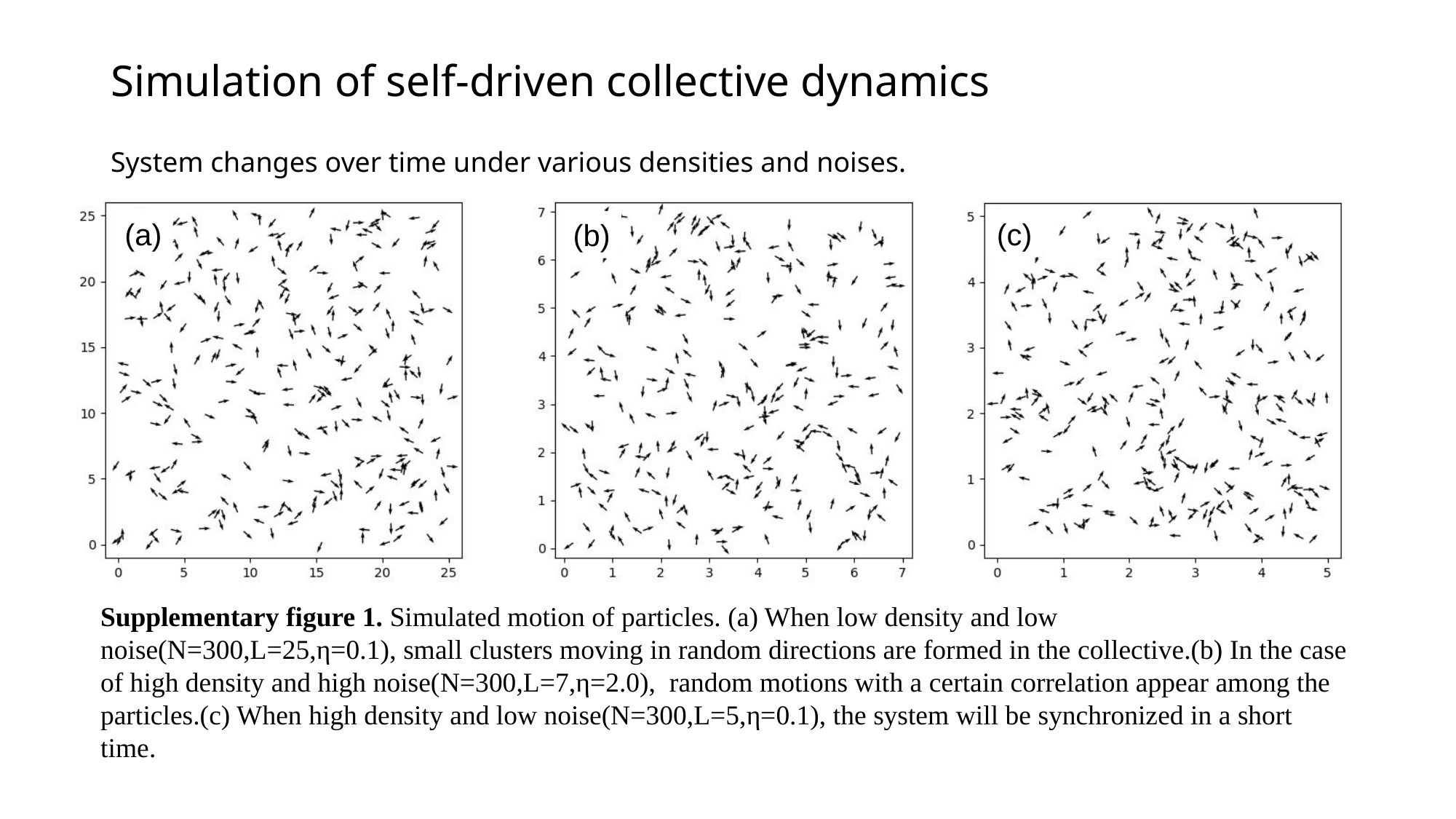

# Simulation of self-driven collective dynamics
System changes over time under various densities and noises.
(a)
(c)
(b)
Supplementary figure 1. Simulated motion of particles. (a) When low density and low noise(N=300,L=25,η=0.1), small clusters moving in random directions are formed in the collective.(b) In the case of high density and high noise(N=300,L=7,η=2.0), random motions with a certain correlation appear among the particles.(c) When high density and low noise(N=300,L=5,η=0.1), the system will be synchronized in a short time.
